# Predictors of failure on second-line antiretroviral therapy with protease inhibitor mutations in Uganda

**DOI:** 10.1186/s12981-021-00338-y

**Published:** 2021-04-21

**Authors:** Hellen Musana, Jude Thaddeus Ssensamba, Mary Nakafeero, Henry Mugerwa, Flavia Matovu Kiweewa, David Serwadda, Francis Ssali

**Affiliations:** 1 College of Health Sciences, School of Public Health, Makerere University, Kampala, Uganda; 2 Joint Clinical Research Centre, Kampala, Uganda; 3 Makerere University-Johns Hopkins University Research Collaboration, Kampala, Uganda; 4 Center for Innovations in Health Africa (CIHA Uganda), Kampala, Uganda; 5 Department of Epidemiology and Biostatistics, Makerere University School of Public Health, Kampala, Uganda; 6 VIVES University of Applied Sciences, Health Care Programs, Kortrijk, Belgium

**Keywords:** Second-line ART failure, Predictors, Protease inhibitor mutations, Uganda

## Abstract

**Introduction:**

Failure on second-line antiretroviral therapy (ART) with protease inhibitor (PI) mutations (VF-M) is on the rise. However, there is a paucity of information on the factors associated with this observation in low-income countries. Knowledge of underlying factors is critical if we are to minimize the number of PLHIV switched to costly third-line ART. Our study investigated the factors associated with VF-M.

**Methods:**

We conducted a matched case–control analysis of patients' records kept at the Joint Clinical Research Center, starting from January 2008 to May 2018. We matched records of patients who failed the second-line ART with major PI mutations (cases) with records of patients who were virologically suppressed (controls) by a ratio of 1:3. Data analysis was conducted using STATA Version 14. Categorical variables were compared with the outcomes

failure on second-line ART with PI mutations using the Chi-square and Fisher's exact tests where appropriate. Conditional logistic regression for paired data was used to assess the association between the outcome and exposure variables, employing the backward model building procedure.

**Results:**

Of the 340 reviewed patients' records, 53% were women, and 6.2% had previous tuberculosis treatment. Males (aOR = 2.58, [CI 1.42–4.69]), and patients concurrently on tuberculosis treatment while on second-line ART (aOR = 5.65, [CI 1.76–18.09]) had higher odds of VF-M. ART initiation between 2001 and 2015 had lower odds of VF-M relative to initiation before the year 2001.

**Conclusion:**

Males and patients concomitantly on tuberculosis treatment while on second-line ART are at a higher risk of VF-M. HIV/AIDS response programs should give special attention to this group of people if we are to minimize the need for expensive third-line ART. We recommend more extensive, explorative studies to ascertain underlying factors.

## Background

Antiretroviral therapy (ART) remains the only scalable biomedical intervention for reducing the impact and effect of HIV/AIDS in Sub Saharan Africa, which disproportionately carries 70% of the global HIV burden [1]. Uganda, whose current HIV prevalence is 6.2% among 15–49 years [2], is among the ten high burden countries that account for almost 80% of all people living with HIV in this region [3]. By 2017, an estimated 1.3 million Ugandans were living with HIV, of whom 67% were on ART [4].

### Second-line antiretroviral therapy

In sub-Saharan Africa, the proportion of HIV positive patients on second-line ART is between 1–5% [5–7] and is expected to rise to 0·5–3·0 and 0.8–4.6 million people between 2020 and 2030 [8]. In Uganda, 3.77% of PLHIV are on second-line ART [4] which, is composed of a PI-based regimen of boosted lopinavir or atazanavir, and a recycled NRTI [9]. The rising number of patients on second-line ART reduces the availability of alternative treatment options in developing countries whose health systems are still dependent on foreign aid to provide ART [10, 11]. Beyond the high cost of second-line regimens, failure on first-line ART is associated with poor adherence [12, 13], which, if not addressed, means that patients initiated on second-line ART are also likely to fail on this regimen. Failure on second-line ART (having two subsequent viral counts of or greater than 1000 copies/ml, done at least 3–6 months apart) means that care providers have to switch such patients to third-line ART [9] which is has a higher pill burden and toxicity [14].

### Failure on second-line antiretroviral therapy

Studies in limited-resource settings have reported second-line ART failure rates of 21.8–33%, 14–38%, 15—38.5%, and 10–38.0% at 6, 12, 24, and 36 months of initiation on second-line ART among adults respectively [15–18]. That said, second-line ART failure is further complicated by major PI mutations.

M46I/L, N88S, V82A/F/T/S/M, I84IV, and I54V/A/S, G48V/M/Q and L76V, which decrease susceptibility to the PIs [19]. The prevalence of second-line ART failure with PI mutations (VF-M) is estimated in the range of 18.5 to 40% and is predicted to rise further as more patients are switched to second-line regimens [18, 20, 21]. However, there is a paucity of information in developing countries on the factors associated with VF-M. The few studies conducted have pointed at age and tuberculosis treatment as factors. For example, in a small cohort of 44 patients, [23] found age as the only factor, whereby patients above 24 years of age were at a higher risk of failing with major PI mutations [22]. Another study among children less than three years of age highlighted the timing of tuberculosis treatment while on second-line ART, and protease inhibitor dosing strategy [23].

More research on the factors associated with VF-M is critical to inform public health experts, HIV/AIDS policymakers, and implementers on how best to minimize the likelihood of having more patients switched to third-line ART. This is given the cost of third-line ART costs eighteen times and seven times higher than the lowest price of first and second-line ART, respectively [14], is more intolerable, and costs more in terms of resources required for its provision [24]. To our knowledge, this study provides the first body of evidence to understand the factors associated with VF-M in the context of developing Uganda.

## Methods

The study was conducted at the Joint Clinical Research Center (JCRC), a high-volume HIV/AIDS health and research care facility located in Wakiso district, Uganda. Currently, the institution takes care of over 15,402 people living with HIV/AIDS (PLHIV), of whom 17% are on second-line ART. We reviewed records of routinely collected clinic data on PLHIV for the period between January 2008 and May 2018. This period coincided with the period when JCRC started conducting viral load monitoring and the period with the most updated information the researchers could get during data collection. We developed a customized data collection tool to capture variables of interest for both cases and their corresponding matched controls. Cases were records of patients who had VF-M. Controls were matches at the time (month and year) the cases occurred (time of genotype). A matching ratio of 1:3 was chosen to increase the power of the study.

### Sequencing for PI mutations

Analysing PI mutations was performed using the Celera Diagnostics ViroSeq HIV-1 Genotyping System (version 2.0). Sequence data were analyzed using the Sequence Analysis software and Celera Diagnostics ViroSeq HIV-1 Genotyping System software (version 2.8). The genotypic results were interpreted for each drug according to the 2011 version of the Stanford algorithm [19].

### Sampling

**Fig. 1:**
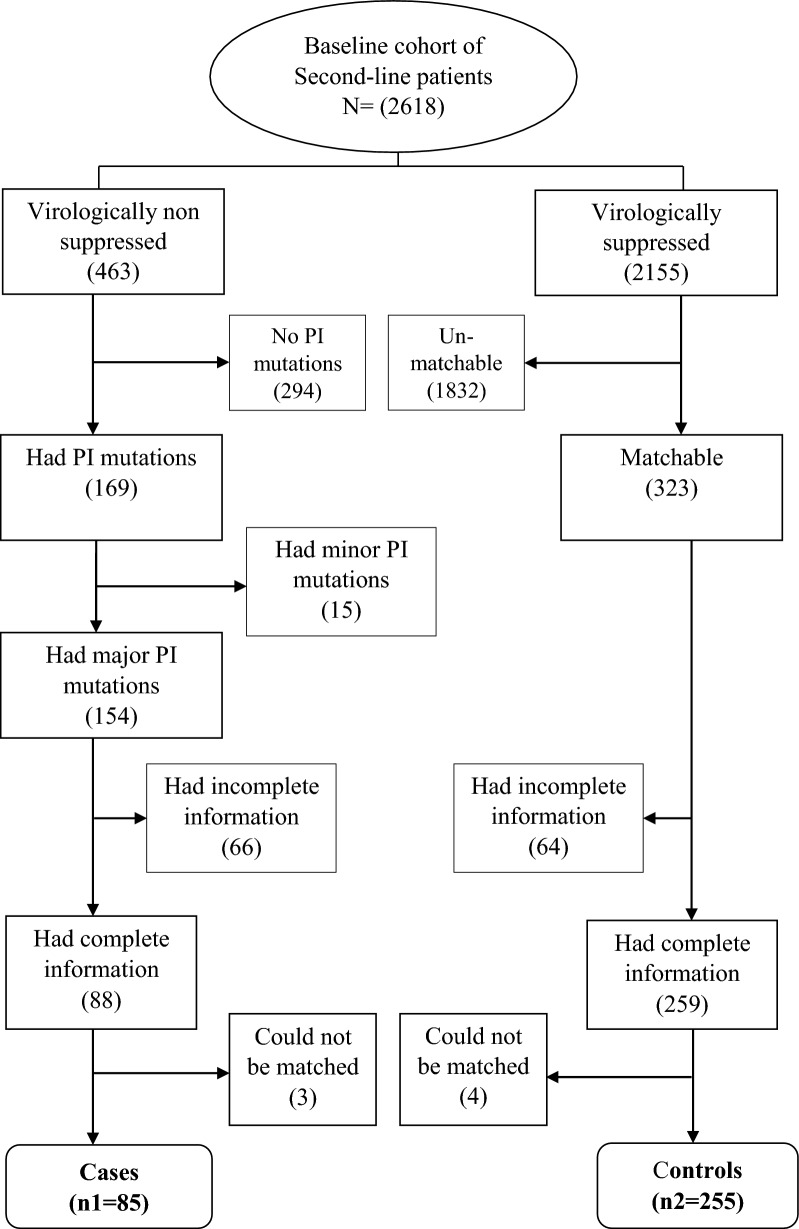
Study sampling strategy

Of the 2,618 records of patients on second-line ART, 2,155 were virologically suppressed. Of the four hundred sixty-three non-suppressed patients, 169 had PI mutations, with major mutations being among 154 patients. Of the 154 records of patients with major PI mutations (cases), we reviewed 85 (49.4%) files since 66 case files were rejected due to incomplete information, and three could not be matched. The cases were matched against 255 controls (See Fig. 1).

### Inclusion and exclusion criteria

For cases, we included all patients who failed on second-line ART between Jan 2008 and May 2018, had complete data and possessed major PI mutations. On the other hand, records of matchable virologically suppressed patients on second-line ART with complete records were controls. Incomplete and unmatchable records were excluded from the analysis.

### Study variables

The dependent variable was failure on second-line ART (≥ 1000 viral copies/milliliter of blood) with major HIV drug-resistant PI mutations (VF-M). Independent variables were age, gender, viral load at the initiation of the second-line of ART, type of first-line and second-line ART regimen, presence of comorbidities, year of ART initiation, and duration on first-line ART and history of tuberculosis management while on second-line ART.

### Data collection

Data were abstracted from hard copy and online patient case management files kept by the JCRC clinic. It was after entered onto a hard copy abstraction tool designed by the researchers. During the data abstraction process, we did not include any patient identifier information. We collected information on patients': age, gender, viral load, and CD4 before initiation of second-line ART, comorbidities while on second-line ART, year of ART initiation, and duration on first-line ART and concomitant treatment for tuberculosis. The patient identifier sheet was kept under key and lock by the researchers.

### Data analysis

The abstracted raw data were double entered into MS Excel and compared to rule out discrepancies. The final dataset was exported to Stata Version 14 (Stata Corp LP, College Station, Texas) software for analysis. Categorical variables were compared with the outcome: failure on second-line ART with PI mutations (VF-M) using the Chi-square test and Fisher's exact test where appropriate. Testing for multicollinearity was done using the variance inflation factors (VIF) approach. Conditional logistic regression for paired data was used to obtain the odds ratios measuring the magnitude of the association between the outcome and the exposure variables. The backward model building procedure was used in which all variables that were significant at the 20% level of significance during simple regression were considered for the multiple regression model. Only factors that were significant at the 5% level were maintained in the final model. Results were reported as the multivariable-adjusted odds ratios with their corresponding 95% confidence intervals (CI) for associations between the exposure variables and the outcome.

### Ethical considerations

Regulatory approval to proceed with the study was sought from the Makerere University Higher Degrees Institutional Review Board (FWA 00011353), and the JCRC administration gave written permission to collect the data. Patient confidentiality was ensured throughout the project's lifetime and after.

## Results

Of the 340 patients' records we reviewed, 53% (n = 179) belonged to women, of whom 33% (n = 28) were cases. The majority (83.2%) of the patients had an NNRTI based regimen for their first-line ART, while 91.8% (n = 312) were on lopinavir for their second-line ART. Only 11% (n = 36) of the study population had comorbidities, the majority of whom (75%) were in the control arm. Relatedly, 6% (n = 21) of the clients were concurrently on second-line ART and TB treatment, of whom the majority (67%) were cases. The median age, CD4 count, and viral load at the start of second-line ART was 38 years, 115 cells, and 67,965 viral copies per ml of blood plasma, respectively. Cases were associated with lower median CD4 counts (68.5) and higher median viral counts (233,700 [p-value ≤ 0.001]) at the start of second-line ART. There was a statistically significant difference in gender (p-value ≤ 0.001), second-line regimens (p-value = 0.001), year of ART initiation (p-value ≤ 0.001) and being on tuberculosis treatment while on second-line ART (p-value ≤ 0.001) among cases and controls (Table 1).

**Table 1:** Study descriptive characteristics

Characteristic	Controls	Cases	Total	p-value
	**n (col %)**	**n (col %)**	**n (col %)**	
*Gender*				
Female	151(59.2)	28(32.9)	179(52.6)	< 0.001
Male	104(40.8)	57(67.1)	161(47.4)	
Total	255(100)	85(100)	340(100)	
*Type of second-line drug*				
lopinavir	241(94.5)	71(83.5)	312(91.8)	0.001
Atazanavir	8(3.1)	4(4.7)	12(3.5)	
Other PI^µ^	6(2.4)	10(11.8)	16(4.7)	
Total	255(100)	85(100)	340(100)	
*TB treatment*^*§*^				
No	246(97.2)	71(83.5)	317(93.8)	< 0.001
YES	7(2.8)	14(16.5)	21(6.2)	
Total	253(100)	85(100)	338(100)	
*Year of ART initiation*				
Before 2001	7(2.7)	23(27.1)	30(8.8)	< 0.001
2001–2005	170(66.7)	41(48.2)	211(62.1)	
2006–2010	64(25.1)	19(22.4)	83(24.4)	
2011–2015	14(5.5)	2(2.4)	16(4.7)	
Total	255(100)	85(100)	340(100)	
*Type of first-line drug*				
NNRTI based	217(85.1)	66(77.6)	283(83.2)	0.111
NON-NNRTI^∞^	38(14.9)	19(22.4)	57(16.8)	
Total	255(100)	85(100)	340(100)	
*Other comorbidities *^*§*^				
No	226(89.3)	76(89.4)	302(89.3)	0.983
Yes	27(10.7)	9(10.6)	36(10.7)	
Total	253(100)	85(100)	338(100)	
*Age at start of 2*^*nd*^* line: median (IQR)*				
	38(32–46)	38(31–46)	38(32–46)	0.300
*CD4 at start of 2*^*nd*^* line: median (IQR)*				
	130(62–250)	68.5(18–181)	115(41–234)	0.079
*VL at start of 2*^*nd*^* line: median (IQR)*^*¥*^				
	43,005.5(13,645.5–135,553.5)	233,700(65,985.5–601,989)	67,965(18,846.5–189,160)	< 0.001
*Duration on 1st line: median (IQR)*				
	4 (2 – 5)	3 (2–6)	3 (2–5)	0.210

^§^Missing data on two observations, ^¥^ Missing data on 128 observations, TB: Tuberculosis, VL: Viral Load, IQR: Interquartile Range, ∞Non-NRTI: triple nucleosides, µ “other PI”: saquinavir and nelfinavir

Table 2 shows that by gender disaggregation, 57 cases (67%) were males, of whom 81% (n = 46) were on lopinavir for their second-line ART. Furthermore, of all patients with comorbidities at the start of second-line ART, 47% (n = 17) of them were males in the control arm. The proportion of female cases who were on TB treatment at the start of second-line therapy was slightly higher (17.9%) compared to male cases (15.8%). However, this could be attributable to lesser numbers of female cases. Relatedly, female cases had a higher median viral count at the start of second-line ART (233,700) compared to their male counterparts (139,901).

**Table 2:** A gender-based comparison of descriptive study characteristics

Characteristic	Females			Males		
	Controls	Cases	Total	Controls	Cases	Total
	n (col %)	n (col %)	n (col %)	n (col %)	n (col %)	n (col %)
*Type of Second-line drug*						
Alluvia	143(94.7)	25(89.3)	168(93.9)	98(94.2)	46(80.7)	144(89.4)
Atazanavir	5(3.3)	3(10.7)	8(4.5)	3(2.9)	1(1.8)	4(2.5)
Other PI^µ^	3(2.0)	0(0)	3(1.7)	3(2.9)	10(17.5)	13(8.1)
Total	151(100)	28(100)	179(100)	104(100)	57(100)	161(100)
*TB treatment* ^ψ^						
No	146(97.3)	23(82.1)	169(94.9)	100(97.1)	48(84.2)	148(92.5)
Yes	4(2.7)	5(17.9)	9(5.1)	3(2.9)	9(15.8)	12(7.5)
Total	150(100)	28(100)	178(100)	103(100)	57(100)	160(100)
*Year of ART initiation*						
Before 2001	6(4.0)	2(7.1)	8(4.5)	1(1.0)	21(36.8)	22(13.7)
2001–2005	91(60.3)	15(53.6)	106(59.2)	79(76.0)	26(45.6)	105(65.2)
2006–2010	44(29.1)	9(32.1)	53(29.6)	20(19.2)	10(17.5)	30(18.6)
2011–2015	10(6.6)	2(7.1)	12(6.7)	4(3.8)	0 (0)	4(2.5)
Total	150(100)	28(100)	179(100)	104(100)	57(100)	161(100)
*Type of First-line drug*						
NNRTI based	129(85.4)	22(78.6)	151(84.4)	88(84.6)	44(77.2)	132(82.0)
NON-NNRTI^∞^	22(14.6)	6(21.4)	28(15.6)	16(15.4)	13(22.8)	29(18.0)
Total	151(100)	28(100)	179(100)	104(100)	57(100)	161(100)
*Other comorbidities*^§^						
No	139(93.3)	28(100)	167(94.4)	87(83.7)	48(84.2)	135(83.9)
Yes	10(6.7)	0(0)	10(5.6)	17(16.3)	9(15.8)	26(16.1)
Total	149(100)	28(100)	177(100)	104(100)	57(100)	161(100)
No	139(93.3)	28(100)	167(94.4)	87(83.7)	48(84.2)	135(83.9)
Yes	10(6.7)	0(0)	10(5.6)	17(16.3)	9(15.8)	26(16.1)
Total	149(100)	28(100)	177(100)	104(100)	57(100)	161(100)
Age at the start of 2nd line ART: median (IQR)	36 (31–41)	35.5(31.5–38)	36(31–41)	43(35–49)	39(31–48)	42(33–49)
CD4 at the start of 2^nd^ line ART: median (IQR)	155(77–253)	65.5(15–135)	137(62–243)	101.5(37–230)	78.5(28.5–205)	93(34–205)
VL at start of 2^nd^ line ART: median (IQR)^¥^	37,706(12,880–135,248)	233,700(51,014–547,922)	50,305.5(1453.5–162,197)	54,588(14,444–135,859)	139,901(72,510–645,978)	76,115(21,919.5–250,702)
*Duration on 1st line: median (IQR)*	4(2–5)	3(1.5–4)	3(2–5)	3(2–5)	4(2–6)	3(2–6)

^§^Missing data on two observations, ^ψ^ Missing data on one observation, ^¥^ Missing data on 128 observations, TB: tuberculosis, VL: Viral Load, IQR: Interquartile Range, ART: Antiretroviral Therapy, ∞Non-NRTI: triple nucleosides, µ “other PI”: saquinavir and nelfinavir

### Factors for failure on second-line ART with PI mutations

Table 3 shows that gender, type of second-line regimen, and tuberculosis treatment while on second-line ART were associated with VF-M at the simple regression stage. Males had higher odds of VF-M compared to females (uOR = 3.09, [CI 1.8–5.31]). Relatedly, patients who had “other” PIs for their second-line ART were more likely to have VF-M (uOR = 5.66, [CI 1.92–16.66]) compared to those who had lopinavir as their second-line regimen. Patients concurrently on tuberculosis treatment, and second-line ART were also more likely to have VF-M compared to colleagues with no tuberculosis treatment at initiation on second-line therapy (uOR = 7.54, [CI 2.69–21.08]). At multiple regression analyses, gender (p = 0.002), being on tuberculosis treatment while on second-line ART (p = 0.004), and year of ART initiation were significantly associated with VF-M. Specifically, males (aOR = 2.58, [CI 1.42–4.69]), patients concurrently on tuberculosis treatment while on second-line ART (aOR = 5.65, [CI 1.76–18.09]) had higher adjusted odds of VF-M. Initiation on ART between 2001 and 2005, 2006 and 2010, 2011 and 2015 had lower adjusted odds of VF-M aOR = 0.06[CI 0.02–0.22], aOR = 0.07[CI 0.02–0.28], and aOR = 0.03, [CI 0.00–0.26] respectively, compared to initiation before 2001. Generally, all three factors were more prevalent among males (Table 2). Multicollinearity among the exposure variables was investigated before the model building, and all of the variance inflation factors (VIFs) were below 10, indicating the absence of multicollinearity.

**Table 3:** Complete case analysis

Characteristic	Unadjusted OR (95% CI)	p-value	Adjusted OR (95% CI)	p-value
*Gender*				
Female	1.00		1.00	
Male	3.09(1.80–5.31)	< 0.001	2.58(1.42–4.69)	0.002*
*Type of second-line drugs*				
lopinavir	1.00		1.00	
Atazanavir	1.59(0.44–5.82)	0.483	3.4(0.79–14.34)	0.101
Other PI^µ^	5.66(1.92–16.66)	0.002	3.92(1.15–13.38)	0.671
*TB treatment*				
No	1.00		1.00	
Yes	7.54(2.69–21.08)	< 0.001	5.65(1.76–18.09)	0.004*
*Year of ART initiation*				
Before 2001	1.00		1.00	
2001–2005	0.05(0.01–0.16)	< 0.001	0.06(0.02–0.22)	< 0.001*
2006–2010	0.05(0.02–0.20)	< 0.001	0.07(0.02–0.28)	< 0.001*
2011–2015	0.02(0.00–0.17)	< 0.001	0.03(0.00–0.26)	0.002*
*Type of 1*^*st*^* line drug*				
NNRTI based	1.00			
NON-NNRTI^∞^	1.81(0.92–3.55)	0.085	–	–
*Other comorbidities*				
No	1.00			
Yes	1.00(0.44–2.26)	1.000	–	–
*Age at start of 2*^*nd*^* line*				
	0.99(0.97–1.01)	0.300	–	–
*CD4 at start of second line*				
	0.99(0.99–1.00)	0.079	–	–
*Duration on 1*^*st*^* line*				
	1.07(0.96–1.18)	0.210		

^¶^ Wide confidence interval due to small sample size ^*^statistically significant at the 5% level, TB: tuberculosis, VL: Viral Load, IQR: Interquartile Range, ART: Antiretroviral Therapy, ∞Non-NRTI: triple nucleosides, µ “other PI”: saquinavir and nelfinavir

## Discussion

Our study showed that the majority of the cases were males (67.1%) (Table 1), and were more likely to have VF-M (p = 0.002). Studies conducted in developing countries have shown that male HIV patients are more likely to present to HIV care facilities with advanced disease as compared to their female counterparts [25, 26]. Moreover, advanced HIV/AIDS is a critical predictor for failure on second-line ART [27, 28]. Furthermore, research has shown that males are more prone to virological failure while on second-line ART than females [29, 30], attributable to poor adherence and higher odds of alcohol consumption while on ART [31, 32]. However, our findings are contrasted by a South African study, which showed that 60% of patients who had VF-M were women [33]. That said, our results continue to highlight male HIV positive patients as a key vulnerable population that needs special attention if we are to maintain them on second-line ART over an extended period.

### Age and VF-M

Chimbetete et al. [22] found that older patients (> 24 years of age) were associated with higher odds of VF-M. This could be attributed to long periods of exposure on first-line drugs, some of which are recycled to form part of second-line regimens[34]. Furthermore, long-term exposure to ART is associated with higher odds of non-adherence [35], which could be the case for older adults. That said, our study did not find any statistical difference between age and VF-M. We recommend more studies to understand the factors behind younger HIV/AIDS patients on second-line ART having lower odds of PI mutations at failure.

### HIV/TB coinfection and VF-M

Concomitant tuberculosis treatment, while on second-line ART, was higher among cases (67%) and significantly associated with VF-M (p ≤ 0.004). Our findings are in line with a study conducted by Rossouw et al. 2015, which found that children on tuberculosis treatment while on second-line ART were more likely to have VF-M [23]. This is attributable to factors such as higher pill burden [36, 37], HIV/TB coinfection being associated with advanced HIV/AIDS [38], and the fact that rifabutin is not readily available in the resource-limited settings as a replacement for Rifampicin which is known to reduce the pharmacokinetic levels of PIs and consequently their efficacy [39, 40]. Our findings highlight VF-M as an outcome of the continued prescription of rifampicin and stress the need for governments in developing countries to adopt rifabutin for HIV/TB co-infected patients. HIV care providers should also provide more personalized attention, counseling, and support to patients concomitantly on TB and second-line ART. This is because they are at a higher risk of VF-M. Furthermore, HIV clinical care specialists have to weigh the options of initiating patients with TB on PIs.

### Year of ART initiation and VF-M

Patients who initiated ART after 2001 were less likely to have VF-M as compared to colleagues who were started on ART before. Patients enrolled on ART between 2001–2005, 2006–2010, 2011–2015 had a 96%, 97%, and 93% lower likelihood of VF-M, respectively, as compared to those initiated on ART before 2001. This is attributed to health systems improvements in the provision of HIV/AIDS care services [41] For example, the introduction of differentiated care models has improved adherence because drugs are taken closer to where patients live. Conversely, before 2001 patients had to return to health facilities within short intervals, which was economically tasking. Relatedly, before 2001, patients on ART had higher pill burdens, which made it hard for them to adhere to treatment [41] as compared to those post-2001 when the improvement in dose formulations was made to reduce pill burdens. Furthermore, due to high levels of primary resistance to NNRTIs, patients initiated on ART of late are given dolutegravir for first-line ART. For this, patients are likely to stay longer on first-line since it’s a new class of drugs with a high genetic barrier to resistance and can be co-formulated, leading to once-daily dosing with tenofovir/lamivudine [42].

### Type of second-line ART and VF-M

Patients who had “other PIs” saquinavir and nelfinavir had lower odds of VF-M compared to counterparts on lopinavir or atazanavir despite the associated higher pill burden of “other PIs” like saquinavir [43] and their low genetic barrier to resistance and reduced bioavailability [44]. The existence of this phenomenon needs further exploration. More to this, it is essential to note that in Uganda, due to increased access to HIV care and reliable national supply of lopinavir and boosted atazanavir, which is the recommended PIs for second-line ART, saquinavir is rarely used except for third-line ART.

### Significance of the study

To our knowledge, this study is the first endeavor to investigate the factors associated with second-line ART failure with PI mutations. The study was conducted within an HIV care provision setting, which reflects ground reality, and our measures were based on WHO standards for assessing failure on second-line ART. Due to existing data management mechanisms for data validity at JCRC, data used for this study was relatively good and reliable. More importantly, we employed a robust data collection system and trained all staff involved in the data collection and management process to minimize errors and ensure the quality and validity of results.

### Study limitations

Our study had a couple of limitations. First, we excluded 69 (45%) files under the “case” arm, and 68 (21%) files under the “control” arm due to data incompleteness, which could have affected the findings. However, we worked with data of over 50% of the whole population of interest, which is generalisable. Second, there might have been original data entry errors since our analysis was solely based on routinely collected project data. Third, our study was conducted within the limitations of case–control studies, with the odds ratios unstable as reflected by the wide confidence intervals. This was minimised by increasing the power of the study. That said, our findings provide a platform for more extensive longitudinal studies to understand further the underlying factors and co-factors for second-line ART failure with PI mutations within low-income settings.

## Conclusion

Our findings provide new insights that male patients, HIV patients co-infected with TB, and patients on PIs such as saquinavir are more likely to fail on second-line ART with PI mutations. It is recommendable that HIV care providers in developing countries design factor specific interventions such as counselling and evaluations to provide targeted additional monitoring and support for those at risk of virological failure.

## Data Availability

The datasets generated and analyzed during the current study are not publicly available due to ethical requirements by the ethics committee. Still, they are available from the corresponding author on a reasonable request.

## Abbreviations

AIDS: Acquired immunodeficiency syndrome
aOR: Adjusted Odds Ratio
ART: Antiretroviral Therapy
CD4: Cluster of Differentiation 4
CI: Confidence Interval
HIV: Human Immunodeficiency Virus
IQR: Interquartile Range
JCRC: The Joint Clinical Research Center
NRTIs: Nucleoside/Nucleotide Reverse Transcriptase Inhibitors
NNRTIs: Non-nucleoside Reverse Transcriptase Inhibitors
PIs: Protease Inhibitors
PLHIV: People Living with HIV/AIDS
TB: Tuberculosis
uOR: Unadjusted Odds Ratio
VIF: Variance Inflation factor
VL: Viral Load

## References

[1] Kharsany AB, Karim QA (2016). HIV infection and AIDS in Sub-Saharan Africa: current status, challenges and opportunities. Open AIDS J.

[2] M.O.H. Uganda population-based HIV impact assessment. 2016, Ministry of Health.

[3] Wu J (2019). HIV/AIDS in Sub-Saharan Africa: to what extent is poverty responsible for the high prevalence. AMSA J Global Health.

[4] Moh U. Uganda HIV/AIDS country progress report July 2016-JUNE 2017. july 2016-June 2017, Ministry of Health Uganda.

[5] Madec Y (2013). Persistent difficulties in switching to second-line ART in sub-Saharan Africa—a systematic review and meta-analysis. PLoS ONE.

[6] Haas AD (2015). Monitoring and switching of first-line antiretroviral therapy in adult treatment cohorts in sub-Saharan Africa: collaborative analysis. The lancet HIV.

[7] Keiser O (2009). Switching to second-line antiretroviral therapy in resource-limited settings: comparison of programmes with and without viral load monitoring. AIDS (London, England).

[8] Estill J (2016). The need for second-line antiretroviral therapy in adults in sub-Saharan Africa up to 2030: a mathematical modelling study. The Lancet HIV.

[9] MoH. The Republic of Uganda. Consolidated guidelines for HIV prevention and treatment in Uganda; 2018.

[10] Olakunde B (2019). Revisiting aid dependency for HIV programs in sub-Saharan Africa. Public Health.

[11] Burrows D et al. Transitions from donor funding to domestic reliance for HIV responses. Recommendations for transitioning countries. Nairobi: APM Global Health; 2016.

[12] Patrikar S (2017). Predictors of first line antiretroviral therapy failure and burden of second line antiretroviral therapy. Med J Armed Forces India.

[13] Ayalew MB (2016). First-line antiretroviral treatment failure and associated factors in HIV patients at the University of Gondar Teaching Hospital, Gondar, Northwest Ethiopia. HIV/AIDS (Auckland, NZ).

[14] Medecins Sans Frontieres (MSF). Untangling the web of antiretroviral price reductions. 2016, Medecins Sans Frontieres (MSF).

[15] Ajose O (2012). Treatment outcomes of patients on second-line antiretroviral therapy in resource-limited settings: a systematic review and meta-analysis. AIDS.

[16] Stockdale AJ (2018). Effectiveness of protease inhibitor/nucleos (t) ide reverse transcriptase inhibitor–based second-line antiretroviral therapy for the treatment of human immunodeficiency virus type 1 infection in sub-Saharan Africa: a systematic review and meta-analysis. Clin Infect Dis.

[17] Win MM (2011). Virologic and immunologic outcomes of the second-line regimens of antiretroviral therapy among HIV-infected patients in Thailand. J Int Assoc Physicians AIDS Care.

[18] Boender TS (2016). Protease inhibitor resistance in the first 3 years of second-line antiretroviral therapy for HIV-1 in sub-Saharan Africa. J Infect Dis.

[19] Stanford University. HIV drug resistance database. 1998–2019, Stanford University,.

[20] Fily F (2018). HIV-1 drug resistance testing at second-line regimen failure in Arua, Uganda: avoiding unnecessary switch to an empiric third-line. Tropical Med Int Health.

[21] Dutta N et al. Virologic failure on anti-retroviral therapy without HIV drug resistance mutation. J Immune Disord Ther 2018;1(2).

[22] Chimbetete C et al. HIV-1 drug resistance and third-line therapy outcomes in patients failing second-line therapy in Zimbabwe. In: Open forum infectious diseases. Oxford University Press US. 2018. 10.1093/ofid/ofy005 PMC5801603 29435471

[23] Rossouw TM (2015). Factors associated with the development of drug resistance mutations in HIV-1 infected children failing protease inhibitor-based antiretroviral therapy in South Africa. PLoS ONE.

[24] Cesar C (2014). Use of third line antiretroviral therapy in Latin America. PLoS ONE.

[25] Lifson AR (2019). Advanced HIV disease among males and females initiating HIV care in rural Ethiopia. J Int Assoc Providers AIDS Care.

[26] Westergaard D (2019). Population-wide analysis of differences in disease progression patterns in men and women. Nat Commun.

[27] Fox MP (2012). Rates and predictors of failure of first-line antiretroviral therapy and switch to second-line ART in South Africa. J Acq Immune Def Syndr.

[28] Tsegaye AT (2016). Predictors of treatment failure on second-line antiretroviral therapy among adults in northwest Ethiopia: a multicentre retrospective follow-up study. BMJ Open.

[29] Penot P (2014). The vulnerability of men to virologic failure during antiretroviral therapy in a public routine clinic in Burkina Faso. J Int AIDS Soc.

[30] Anude CJ (2013). Immuno-virologic outcomes and immuno-virologic discordance among adults alive and on anti-retroviral therapy at 12 months in Nigeria. BMC Infect Dis.

[31] Kazooba P (2018). Virological failure on first-line antiretroviral therapy associated factors and a pragmatic approach for switching to second line therapy–evidence from a prospective cohort study in rural South-Western Uganda, 2004–2011. Pan Afr Med J.

[32] Negash T, Ehlers V (2013). Personal factors influencing patients' adherence to ART in Addis Ababa, Ethiopia. J Assoc Nurses AIDS Care.

[33] Moorhouse M (2019). Third-line antiretroviral therapy program in the South African public sector: cohort description and virological outcomes. J Acq Immune Def Syndr.

[34] Suaysod R (2015). Treatment failure in HIV-infected children on second-line protease inhibitor-based antiretroviral therapy. Clin Infect Dis.

[35] Bukenya D (2019). What causes non-adherence among some individuals on long term antiretroviral therapy? Experiences of individuals with poor viral suppression in Uganda. AIDS Res Ther.

[36] Daftary A, Padayatchi N, O'Donnell M (2014). Preferential adherence to antiretroviral therapy over tuberculosis treatment: a qualitative study of drug-resistant TB/HIV co-infected patients in South Africa. Glob Public Health.

[37] Gebremariam MK, Bjune GA, Frich JC (2010). Barriers and facilitators of adherence to TB treatment in patients on concomitant TB and HIV treatment: a qualitative study. BMC Public Health.

[38] Organization, W.H., Guidelines for managing advanced HIV disease and rapid initiation of antiretroviral therapy, July 2017. 2017. 29341560

[39] Semvua HH (2015). Pharmacological interactions between rifampicin and antiretroviral drugs: challenges and research priorities for resource-limited settings. Ther Drug Monit.

[40] Burger D (2006). Effect of rifampin on steady-state pharmacokinetics of atazanavir with ritonavir in healthy volunteers. Antimicrob Agents Chemother.

[41] Grimsrud A (2017). Evidence for scale up: the differentiated care research agenda. J Int AIDS Soc.

[42] Simiele M (2017). UPLC–MS/MS method for the simultaneous quantification of three new antiretroviral drugs, dolutegravir, elvitegravir and rilpivirine, and other thirteen antiretroviral agents plus cobicistat and ritonavir boosters in human plasma. J Pharm Biomed Anal.

[43] López-Cortés LF (2010). Efficacy, safety and pharmacokinetic of once-daily boosted saquinavir (1500/100 mg) together with 2 nucleos (t) ide reverse transcriptase inhibitors in real life: a multicentre prospective study. AIDS Res Ther.

[44] Dandache S (2008). PL-100, a novel HIV-1 protease inhibitor displaying a high genetic barrier to resistance: An in vitro selection study. J Med Virol.

